# Production losses from morbidity and mortality by disease, age and sex in Norway

**DOI:** 10.1177/14034948231188237

**Published:** 2023-07-28

**Authors:** Jonas Minet Kinge, Astrid de Linde, Joseph L. Dieleman, Stein Emil Vollset, Ann Kristin Knudsen, Eline Aas

**Affiliations:** 1 Department of Health Management and Health Economics, University of Oslo, Norway; 2 Centre for Disease Burden, Norwegian Institute of Public Health, Norway; 3 Institute for Health Metrics and Evaluation, USA; 4 Division of Health Services, Norwegian Institute of Public Health, Norway

**Keywords:** Production loss, disease burden, Norway, cost of illness

## Abstract

**Aim:**

The inclusion of production losses in health care priority setting is extensively debated. However, few studies allow for a comparison of these losses across relevant clinical and demographic categories. Our objective was to provide comprehensive estimates of Norwegian production losses from morbidity and mortality by age, sex and disease category.

**Methods:**

National registries, tax records, labour force surveys, household and population statistics and data from the Global Burden of Disease were combined to estimate production losses for 12 disease categories, 38 age and sex groups and four causes of production loss. The production losses were estimated via lost wages in accordance with a human capital approach for 2019.

**Results:**

The main causes of production losses in 2019 were mental and substance use disorders, totalling NOK121.6bn (32.7% of total production losses). This was followed by musculoskeletal disorders, neurological disorders, injuries, and neoplasms, which accounted for 25.2%, 7.4%, 7.4% and 6.5% of total production losses, respectively. Production losses due to sick leave, disability insurance and work assessment allowance were higher for females than for males, whereas production losses due to premature mortality were higher for males. The latter was related to neoplasms, cardiovascular disease and injuries. Across age categories, non-fatal conditions with a high prevalence among working populations caused the largest production losses.

**Conclusions:**

**The inclusion of production losses in health care priority debates in Norway could result in an emphasis on chronic diseases that occur among younger populations at the expense of fatal diseases among older age groups.**

## Introduction

The measurement and valuation of production losses remains a debated topic. Many countries, including Norway, omit them from priority setting in health care. The argument is that it would interfere with equity between disease groups, by emphasizing the working age population [1–4]. Conversely, it has been argued that omitting production losses may give a partial picture of the economic implications of a treatment strategy [5]. A calculation of differences in production losses across disease categories can demonstrate which diseases have the largest potential for reducing these losses.

The aim of this study was to provide estimates of the production loss from morbidity and mortality in Norway and to measure how this production loss was distributed across disease categories, age, sex, and type of production loss.

## Methods

We applied a human capital approach (HCA), which assigns value to a person’s labour participation equal to their work earnings [2,6]. Accordingly, the present value of the future stream of gross earnings becomes the production loss to society of a person’s absence from work [7,8].

### Data

The data used for this study were based on databases covering the full Norwegian population. Data on total person-days of doctor-certified sick leave, total number of work assessment allowance (AAP)-recipients and total number of disability insurance (DI)-recipients were derived from Statistics Norway, while the distributions according to diagnosis, age and sex were provided by the Norwegian Labour and Welfare Administration [9–11]. All data were for 2019, except for the total number of DI-recipients according to diagnosis, age and sex, which were collected for 2017. In this case, the total number of DI-recipients by age–sex matched the 2019 figures; however, the distribution of diseases within each age–sex group was based on 2017 data. Data on the total number of deaths according to age, sex and diagnosis were from the GBD study for Norway in 2019 [12,13].

We used the aggregated data with national coverage to estimate production losses for 12 disease categories, 38 groups for age and sex, and four causes of production loss (sick leave; AAP; DI and mortality). The 38 groups for age and sex used in this study included 19 age groups for each sex, including younger than one year, 1–4 years, 5–9 years, . . . 85 and above. The underlying diseases included in each of the disease categories were overlapping across data sources with one exception: for the sick leave data, the category for communicable diseases also included some general/unspecified codes.

We defined gross wages to include all wages and salaries, bonuses and variable additional allowances in accordance with classifications from Statistics Norway [14]. Following Bugge et al. [8], we defined the surcharge to encompass holiday pay, service pension, employer’s tax, insurance and profit, and set the surcharge to 40% of the wage.

### Estimation strategy

Production losses were calculated for four subcomponents: (a) short-term absence in the form of sick leave, (b) medium-term absence in the form of AAP, (c) long-term disability in the form of DI, and (d) premature mortality (see Supplemental material Table I online for definitions and Norwegian terms). The estimation process proceeded in two steps. First, we estimated the number of episodes of sick leave, AAP, DI and premature mortality by age, sex and disease category. Second, we estimated the production loss of a single episode, which we multiplied with the number of episodes by age–sex, type and disease category (see Supplemental Methods for calculations of production losses for each episode).

## Results

In total, this study included 26,703,846 days of sick leave, 169,046 AAP-recipients, 364,005 DI-recipients and 41,386 deaths in 2019. The total production loss was estimated at NOK371.6bn.

**Figure 1.**
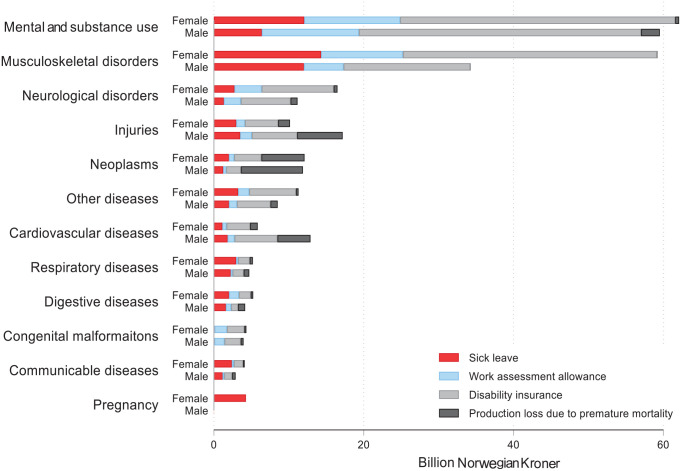
Production loss in Norway by type of loss, disease category and sex, 2019.

Among the 12 disease categories, the highest production losses were for mental and substance use disorders: NOK121.6bn or 32.7% of the total production loss (Table I; Figure 1). Musculoskeletal disorders, which included low back and neck pain, constituted 25.2%, and was the second highest category. Production losses due to sick leave, DI and AAP were higher for females than for males, whereas production losses due to premature mortality were higher for males.

**Table I.** Production loss by disease group, age and type of loss, in 2019.

Disease category	Production loss, 2019 Billion Norwegian kroner	% of total	Per cent by age group (% by columns)		Per cent by cause of production loss (% by columns)			
			Below 45	45 and above	Sick leave	AAP	DI	Premature mortality
Mental and substance use	121.6	32.7	43.5	27.1	22.1	41.8	39.3	8.2
Musculoskeletal disorders	93.5	25.2	14.3	30.8	31.5	26.3	26.8	0.4
Neurological disorders	27.7	7.4	8.6	6.8	4.9	9.6	8.6	3.9
Injuries	27.3	7.4	8.8	6.6	7.8	4.4	5.5	20.8
Neoplasms	24.0	6.5	3.5	8.0	3.9	1.8	3.0	38.2
Other diseases	19.8	5.3	4.1	6.0	6.3	4.1	5.7	3.5
Cardiovascular diseases	18.7	5.0	1.7	6.8	3.5	2.4	4.7	14.8
Respiratory diseases	9.9	2.7	2.6	2.7	6.2	0.8	1.6	3.1
Digestive diseases	9.4	2.5	2.9	2.3	4.3	3.3	1.3	3.4
Congenital malformations	8.3	2.2	4.6	1.0	0.2	4.9	2.4	1.8
Communicable diseases	7.0	1.9	2.1	1.8	4.3	0.8	1.2	1.8
Pregnancy	4.3	1.2	3.3	0.0	5.1	0.0	0.0	0.0
Total	371.6	100	100	100	100	100	100	100

The underlying diseases included in each of the disease categories were overlapping across data sources. The one exception is for the sick leave data, for which the category for communicable diseases also included some general/unspecified codes.

AAP: work assessment allowance; DI: disability insurance

**Figure 2.**
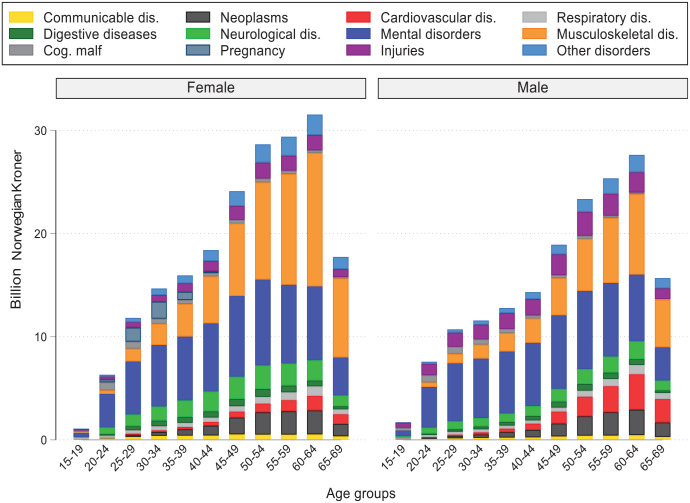
Production loss in Norway by age, sex and disease category, 2019. The underlying diseases included in each of the disease categories were overlapping across data sources. The one exception is for the sick leave data, for which the category for communicable diseases also included some general/unspecified codes. dis.: diseases; Cog. malf: congenital malformations

Production losses increased by age from 0 to 64 years, which was caused by increased DI associated with musculoskeletal disorders for females, and premature mortality for neoplasms and cardiovascular disease for males (Figure 2; Supplemental Figures 1, 2 and 3).

## Discussion

The main causes of production losses in 2019 in Norway were mental and substance use disorders, followed by musculoskeletal disorders, neurological disorders, injuries and neoplasms. Production losses for females were higher than production losses for males and increased by age up until retirement age.

The findings presented in this study are comparable to earlier findings from Norway in 2013, though the earlier version was constructed to measure only losses going beyond own consumption. Hence, the methods applied differ [1]. This study applies HCA, that is, gross earnings, to value production losses [2,7], while Kinge et al. [1] included forgone tax revenue. Consequently, the magnitude of the total loss differs, but the relative distribution by disease categories is comparable.

Although this study did not directly address whether production losses should be adopted by policy makers, it clearly demonstrates the existence of substantial differences across disease categories. A high aggregate disease burden does not in itself warrant high expenditures on prevention and treatment of the disease, as this depends on the cost-effectiveness of interventions in the area. A high aggregate disease burden is instead frequently used to justify resources aimed at disease-specific research or for general institutional policies [15,16].

This study followed Drummond et al. [2] and used a general wage rate, rather than an age-, sex- and disease-specific one. This was done to mitigate equity concerns and applying differential wage rates could have shifted the production losses towards diseases more common in high income groups [17]. As a consequence, diseases with the highest losses are also more prevalent in low socioeconomic groups compared with higher socioeconomic groups [18,19].

This study has limitations that need to be taken into account. First, the magnitude of the production loss depends on the method. HCA might overestimate the production losses, especially in an economy with less than full employment [6]. Second, the estimates do not include production losses from informal care, which might vary substantially by type of diagnosis [20]. Third, the estimates of the economic losses also do not include the reduction in future consumption when illnesses lead to premature deaths. Fourth, we do not include self-certified sick leave, due to missing information about diagnosis. In 2019, the countrywide sickness absence rate in Norway for self-certified sick leave was 3.6% while for doctor-certified leave it was 19.6% [21]. This will lead to an underestimation of the production loss. Finally, our specification does not allow for wage growth over time. This treatment of real wages as static may underestimate losses from premature mortality.

## Conclusions

Our estimate of NOK371.6bn in lost production represents a substantial cost to Norway’s economy. This loss is equivalent to approximately 10.5% of Norway’s GDP in 2019, and is close to the total health expenditure, NOK375.5bn, in that same year [13,22].

The inclusion of production losses in health care priority debates in Norway could result in an emphasis on chronic diseases that occur among younger populations, at the expense of fatal diseases among older age groups.

## Supplemental Material

sj-docx-1-sjp-10.1177_14034948231188237 – Supplemental material for Production losses from morbidity and mortality by disease, age and sex in Norway Supplemental material, sj-docx-1-sjp-10.1177_14034948231188237 for Production losses from morbidity and mortality by disease, age and sex in Norway by Jonas Minet Kinge, Astrid de Linde, Joseph L. Dieleman, Stein Emil Vollset, ann Kristin Knudsen and Eline Aas in Scandinavian Journal of Public Health
