# Supplementary material for: Production losses from morbidity and mortality by disease, age and sex in Norway
Source: Scand J Public Health. 2023 Jul 28;52(6):779–83. doi: 10.1177/14034948231188237 (PMC11308283; doi:10.1177/14034948231188237)
Supplement: sj-docx-1-sjp-10.1177_14034948231188237 – Supplemental material for Production losses from morbidity and mortality by disease, age and sex in Norway [file sj-docx-1-sjp-10.1177_14034948231188237.docx]

**Appendix**

*Appendix to: Kinge et al., Production losses from morbidity and mortality by disease, age, and sex in Norway*

**Content**

*Supplemental Methods*

*Supplemental Table 1: Key Terms*

*Supplemental Figure 1: Production loss due to mortality by age, sex, and disease in 2019*

*Supplemental Figure 2: Production loss due to non-fatal health loss by age, sex, and diseases*

**Supplemental methods**

Sick leave

We assumed that production per person-day was equal to the average gross wage per working day. This was calculated by dividing the mean yearly gross wages by 230 days, which is the number of working days per year for a full-time employee, and adding the 40% surcharge (1, 2).

Work assessment allowance & disability insurance

Work assessment allowance (AAP) is based on each individual’s employment income in the calendar year before their ability to work was reduced by at least 50%. The AAP amounts to 66% of that income, up to NOK 599 148 (3). The DI amounts to 66% of the gross wages, according to the three best years in the five year period prior to illness, up to NOK 599 148 (4) . We estimated the production loss per AAP-recipient and DI-recipient as the mean AAP and DI transfers received by the recipient, by sex, multiplied by 0.66 and the 40% surcharge (5). Technically the AAP and DI transfers are not production losses as they are transfers, but we used them as an estimate of the forgone wages due to disability.

Premature mortality

To estimate the production loss from premature mortality we first estimated lost workdays due to death at each age, by sex, based on life tables, age-sex specific working hours and employment rates from Statistics Norway. Second, we estimated the lost wages due to death, by age and sex, by multiplying the lost workdays with average gross wages from Statistics Norway, including the 40% surcharge (6). We thus assume that wages in the future will be identical to current wages, but discount future wages with an annual rate of 4% according to guidelines from the Norwegian Ministry of Finance (6).

Supplemental Table 1: Key Terms

The following table provides a brief description of each production loss cause for which the Norwegian Labour and Welfare Administration (NAV) provides substitute income. Generally, prior participation in the National Insurance Scheme is a baseline requirement for eligibility.

| Term (in Norwegian) | English Translation | Description |
| --- | --- | --- |
| Uføretrygd | Disability insurance (DI) | DI covers individuals who, because of injury or illness, have a partial or totally reduced ability to work. (4) |
| Arbeidsavklaringspenger (AAP) | Work assessment allowance | AAP is provided to individuals whose ability to work is reduced by at least 50% from injury or illness, given that their future ability to work can be bettered, through treatment or training for example. (3) |
| Sykepenger | Sick leave | Sick leave is covered by an individual’s employer for the first 16 days of illness and is afterwards financed by NAV. Eligibility requirements, among other conditions, include work absence of at least 20% due to illness/injury. (7) |

**Supplemental Figure 1: Production loss due to mortality by age, sex, and disease in 2019**

**

**Supplemental Figure 2: Production loss due to non-fatal health loss by age, sex, and diseases**

1. Statistics Norway. Earnings: Statistics Norway,; 2019 [Available from: <https://www.ssb.no/en/arbeid-og-lonn/lonn-og-arbeidskraftkostnader/statistikk/lonn>.

2. The Norwegain Tax Adeministration. Tax for individuals: The Norwegain Tax Adeministration; 2022 [Available from: <https://www.skatteetaten.no/en/person/taxes/tax-return/find-item/3/2/8/>.

3. Norwegian Labour and Welfare Administration. AAP in brief: Norwegian Labour and Welfare Administration,; 2023 [Available from: <https://www.nav.no/aap/en>.

4. Norwegian Labour and Welfare Administration. About disability benefit: Norwegian Labour and Welfare Administration [Available from: <https://www.nav.no/uforetrygd/enNorwegian>.

5. Statistics Norway. Work assessment allowance 2019 [Available from: <https://www.ssb.no/en/sosiale-forhold-og-kriminalitet/trygd-og-stonad/statistikk/arbeidsavklaringspenger>.

6. Finansdepartement DK. Prinsipper og krav ved utarbeidelse av samfunnsøkonomiske analyser mv. Online] Tilgjengelig på< <https://www>. regjeringen. no/globalassets/upload …; 2014.

7. NAV. Work assessment allowance (AAP) [updated March 16, 2023. Available from: <https://www.nav.no/aap/en>.
